# Exploring indigenous knowledge and practices of the Gurage community on the biosystematics and utilization of Enset landraces for bone fracture and regeneration: the case of Gurage Zone, central Ethiopia region

**DOI:** 10.3389/fphar.2025.1563898

**Published:** 2025-04-28

**Authors:** Temesgen Negassa, Asfaw Meressa, Negera Abdissa, Sileshi Degu, Getachew Addis, Eyob Debebe, Negessa Abdisa, Samuel W/kidan, Dereilo Bekere Belitibo, Sintayehu Ashenef, Werku Shanko, Zeynu Zuber, Lemessa Kumsa, Mewded Kassahun, Frehiwot Teka Assamo, Milkyas Endale

**Affiliations:** ^1^ Traditional and Modern Medicine Research and Development Directorate, Armauer Hansen Research Institute, Addis Ababa, Ethiopia; ^2^ Department of Public Health, College Health Sciences, Ethiopian Defense University, Bishoftu, Ethiopia; ^3^ Department of Orthopedic Surgery Alert Hospital, Addis Ababa, Ethiopia; ^4^ Department of Applied Biology, School of Applied Natural Science, Adama Science and Technology University, Adama, Ethiopia

**Keywords:** Enset, indigenous knowledge, landraces, bone fracture, astara, guarye, kibinar, dere

## Abstract

**Background:**

Enset (*Ensete ventricosum*) is a crucial perennial crop in Ethiopia for both food and medicine. The indigenous knowledge and practices of the use and biosystematics of the different Enset landraces are deeply rooted in the community. Enset corms, traditionally used for bone fracture treatment, are rich in phosphorus, potassium, zinc, and calcium supporting bone healing and mineralization. Thus, the study aims to explore the traditional knowledge and practices of the Gurage community regarding Enset folklore biosystematics and the utilization of Enset in bone healing.

**Methods:**

The study utilized semi-structured interviews, focus group discussions, key informant interviews, and field observations to document traditional medicinal uses, cultivation practices, and indigenous biosystematics of Enset in four selected districts or Woredas (Cheha, Ezhe, Enor, and Gumer) of Gurage Zone, Central Ethiopia from 603 respondents. Descriptive statistics were employed for data presentation.

**Results:**

A total of 37 Enset landraces were identified across the study Woredas. Landraces were identified primarily based on leaf and pseudostem color (62%) and size (24.7%). The majority of respondents (57.7%) were knowledgeable about 6–10 Enset landraces, while 21% identified 11–15 varieties. Four landraces Astara (31.3%), Kibinar (22.9%), Dere (22.4%), and Guarye (20.1%)were most commonly used for healing fractured and broken bones. Corms are the major parts of Enset used for healing fractured bones and setting broken bones by mixing them with yogurt, milk, or meat. Enset also serves as livestock fodder (42.3%) and for making household materials (23.3%), with 91.4% cultivated in home gardens. Disease susceptibility, insects, and wild animals were identified as major challenges for Enset production.

**Conclusion:**

Indigenous knowledge has played a significant role in identifying, classifying, and cultivating Enset landraces. Astara, Kibinar, Dere, and Guarye are the most frequently used Enset landraces for healing fractured and broken bones. Further experimental studies to validate the ethnopharmacological uses of Enset for bone healing are highly recommended.

## Introduction

Numerous crop plants that have been cultivated for thousands of years and played a vital role in the sustenance of local communities remain largely unfamiliar, if not entirely obscure, beyond the regions where they are cultivated and utilized. The food and nutrition security of small-scale farming households is greatly improved by these crops, which are mostly grown in subsistence farming systems in a number of developing nations, including Ethiopia (Morrow et al., 2023). *Ensete ventricosum* (Welw.) Cheesman (family: Musaceae), locally called Enset, one of the earliest developed herbaceous, monocarpic, banana-like crops, is central to Ethiopia’s food security (Sahle et al., 2018). Native crops like Enset are crucial not only for improving food security and nutrition, particularly in subsistence farming communities but also for medicinal purposes. It is estimated that over 20% of Ethiopians depend on Enset for food, medicine, building materials, fiber, and animal feed (Brandt et al., 1997; Borrell et al., 2020).

The Enset farming system (EFS) entails cultivating Enset as a perennial plantation within homestead rings, along with other partner crop species cultivated in the main agricultural lands. This system supports numerous crop types, both intra-specific and inter-specific. The indigenous knowledge of folk biosystematics of Enset landraces and their utilization for medicinal purposes were deeply embedded in the community. Due to changes in the environment and domestication processes affected by indigenous cultures, knowledge, and traditions, the variety of Enset species and associated crop species found in EFS has changed over the ages (Tsegaye and Struik, 2002). Documenting and leveraging local knowledge on classification, managing, and utilizing for medicinal purposes of different Enset landraces is widely recognized as crucial for enhancing farming systems, safeguarding agricultural biodiversity from loss, and recording their therapeutic roles.

**FIGURE 1:**
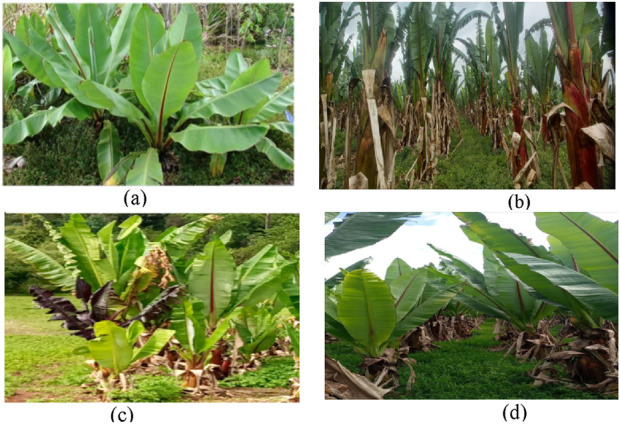
*E. ventricosum* landraces used as traditional medicine ((Astara **(A)**, Kibinar **(B)**, Guarye **(C)**, and Dere **(D)**), Gurage Zone, south central Ethiopia.

Certain landraces of *E. ventricosum* (Figure 1) are believed to possess medicinal properties that are beneficial for treating various human ailments such as weakness, diabetes, and kidney stones, and aiding childbirth (Diana and George, 2013; Mali and Bhadane, 2008). The Tayo Enset landrace’s boiling corm and starchy powder, called bulla, when combined with milk, has been used in the Bonga region to treat conditions like fractures, joint displacement, edema, and shattered bones (Tsehaye and Kebebew, 2006). The Enset variety Sweete is highly recommended in the Areka region for the treatment of bone issues (Daba and Shigeta, 2016). Various Enset landraces have variable starch yields and nutrient contents, however, processed Enset products like kocho, bulla, and corm have been noted to be high in carbs and serve as crucial mineral sources. Kocho is a fermented starch prepared as bread from the corm and pseudostem of Enset, while bulla is a dehydrated result of the juice obtained from the corm grating and pseudostem decortication. Amicho is an unfermented, stripped corm of an immature Enset plant that is boiled, like a potato tuber, before consumption (Brandt et al., 1997). According to reports (Diana and George, 2013), pseudostem and seeds from the allied plant *Ensete superbum* are used to help with delivery and treat conditions including diabetes and kidney stones in humans.

After giving birth, women are given butter and milk-based products called Amicho (unfermented, boiled pieces of corm of young Enset plant) of the Choro clone in Bonga (Tsehaye and Kebebew, 2006) and Asikala in Sidama zone (Assefa and Fitamo, 2016) to encourage placenta discharge. Dairy cows are also administered the Amicho of both Enset clones, combined with salt, to treat comparable conditions (Tsehaye and Kebebew, 2006). Earlier studies have investigated the general traditional herbal medicinal practices in the Gurage zone to document current knowledge being practiced by the community (Teka et al., 2020). Equally important, but less researched and not systematically documented, is specifically, the way the local people classify and benefit from Enset diversity and medicinal uses, especially for treating bone fractures, in the Gurage zone.

Enset corms, widely used in traditional bone fracture treatments, exhibit notable variation in their proximate and mineral compositions across different landraces in Ethiopia. A study in the Gurage Zone (Nuraga et al., 2019) found moisture content ranging from 65.3% to 71.94%, crude fiber from 2.38% to 4.43%, with the Amerat landrace having the highest fiber content. Crude protein levels ranged from 2.42% to 4.74%, while carbohydrate content remained consistent across landraces. Enset corms were found to have higher calcium (97.3–114.3 mg g^−2^) and phosphorus levels compared to other root crops, but lower protein and iron content. They are particularly rich in potassium, with levels reaching 1,654.2 mg g^−2^. A study in Central Ethiopia (Penido and Alon, 2012) also revealed variations in phosphorus, potassium, and zinc levels across landraces. The Astara and Guarye landraces showed the highest phosphorus content (127.41 mg g^−2^), suggesting their potential in bone healing due to phosphate’s role in bone mineralization. Zinc content was highest in the Astara landrace (8.52 mg g^−2^), supporting its use in traditional bone treatments (Kaya et al., 2015; Lin et al., 2018). The high arginine content in enset has been linked to its role in collagen formation and wound healing (Tamrat et al., 2020). Phytochemical analysis showed the Kibnar landrace had the highest tannin content (153.94 mg g^−2^), which may contribute to its traditional use in early-stage bone healing due to tannins’ antimicrobial and anti-inflammatory properties (Haslam, 1996). Phytate levels in enset corms ranged from 149.33 to 195.15 mg g^−2^, suggesting mineral-binding and antioxidative benefits (Hiwot, 2015). Despite the numerous uses of *Ensete ventricosum* in food, medicine, and other applications, comprehensive scientific studies on the species remain insufficient.Preliminary phytochemical screenings have identified several bioactive compounds, such as alkaloids, flavonoids, steroids, quinones, saponins, tannins, and glycosides (Dubiwak et al., 2021), but no detailed quantitative profiles or isolated bioactive compounds have been reported. Therefore, the present study was undertaken with the objective of 1) investigating folk-biosystematics of Enset landraces 2) documenting socio-cultural, ethnomedicinal, and other related uses of Enset 3) identifying parts used for healing fractured and broken bones and modes of preparation (iv) exploring the threatening factors of Enset cultivation.

## Materials and methods

### Study area

The study was conducted in the Gurage zone, South Central region located in south central Ethiopia at 7° 40′0″–8° 30′0″N and 37° 50′0″–38° 40′0″E with an altitudinal range stretching between 1,000 and 3,600 m a. s. l. and covers an area of 5,893.5 km^2^ (Teka et al., 2020; Sahle and Saito 2021. The Zone is composed of 15 Woredas, which are organized into kebeles, which are the smallest administrative units. The people belonging to the Western Gurage Zone speak Amharic, Gurage language, Kebena, and Libido languages. Based on the recent classification of potential vegetation types as described in the literature (Friis et al., 2010), the study area is dominantly characterized by the dry evergreen Afromontane Forest and grassland complex (the undifferentiated Afromontane Forest subtype).

Enset is the main food crop together with *Hordeum vulgare* L. Waif. (barley), pulses, potatoes, and cabbage in the Woreda and at large in the Gurage zone. The major cash crops are *Catha edulis* Forsk (Khat), *Coffea arabica* (Buna), *Eragrostis tef* (Zucc.) Trotter (Teff), and *Guizotia abyssinica* (L. f.) Cass (Noug).

### Informant selection and sample size determination

**FIGURE 2:**
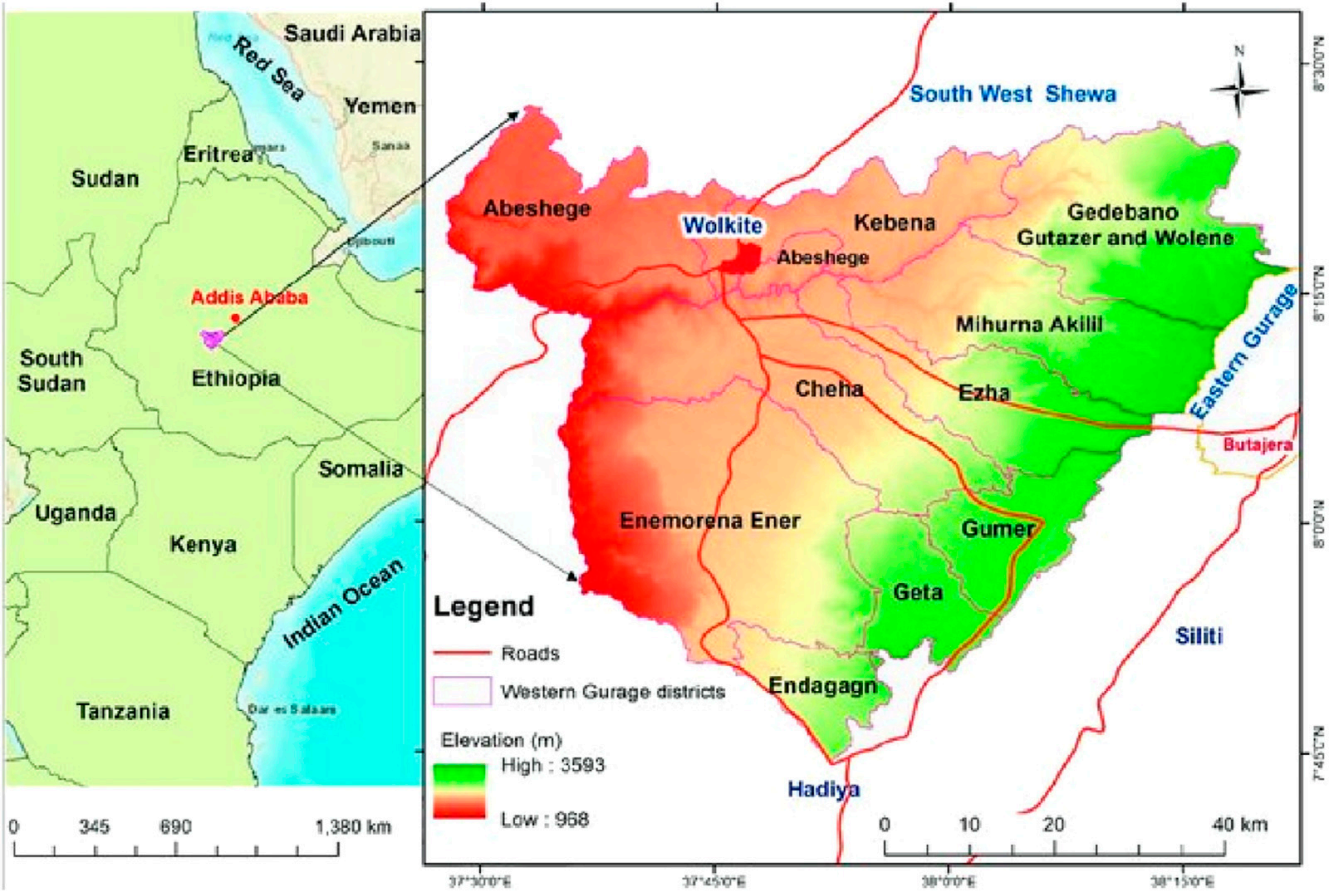
Map of Ethiopia and the study districts in the respective zones.

Among the 15 Woredas in the Gurage zone, 4 Woredas (Cheha, Ezhe, Enor, and Gumer) (Figure 2) were selected purposely for the current study, due to their high population size and wide utilization of Enset. Moreover, from each Woreda, 3 kebeles (totaling 12 kebeles) were selected based on the recommendations from development agents and agricultural officers in association with Enset cultivation, suitability of agroecology, and elevational ranges. A total of 603 informants were selected following (Gomez-Beloz, 2002; Mitiku et al., 2024). Among the 603 informants, 498 were general informants selected randomly from resident booklets and the rest 105 informants were selected purposely based on the recommendations from elders, development agents, and agricultural officers (Table 1).

**TABLE 1:** Sample size distribution for the selected kebeles.

Woreda	Kebele	Number of respondents	Number of respondents	
			General informants	Key informants
Cheha	Moche	60	50	10
	Indibir	31	26	5
	Fereziye	41	33	8
Enor	Agata	63	52	11
	Amogera	51	42	9
	Kucher	34	27	7
Gumer	Agena	37	30	7
	Shebreden	55	45	10
	Wayiredeba	73	60	13
Ezhe	Arekit	18	15	3
	Abeke	44	37	7
	Badnawigor	96	81	15

### Data collection

Before data collection, a permit letter was obtained from the respective Woredas and Gurage administrative offices for conducting the research. Prior informed consent was obtained from each informant before every interview. Data were collected through a combination of methodologies for the acquisition of local knowledge, including literature review, focus group discussions (FGD), in-depth individual interviews, expert elicitations, and observations of cultivation techniques (Walker et al., 1997). A literature review provided the necessary background context of Enset farming systems and cultural links to the farming communities of the Gurage zone. The present status of Enset agriculture and diversity in the Gurage zone was reviewed from several published and unpublished sources and reports. Focus Group Discussion (FGD), semi-structured interviews, and guided field walks were used in the acquisition of the data. FGDs (one group comprising 5–10 members) were held in each of the selected kebeles involving members from local administration, community elders, key informant farmer groups, and other members of participating communities, and full consent of collaboration based on the principle of Free Prior Informed Consent was granted (Perrault, 2004). The FGD was used to allow the identification of participants’ convergent discourses in data collected, to pertaining the knowledge and specific information of Enset varieties. Moreover, ethnomedicinal information of different Enset landraces and parts used in treating bone fractures and broken bones were documented. Individual interviews were carried out together with trained enumerators, who are development agents (DAs) working closely with the communities in the respective selected kebeles. Open questions and free-listing approaches were followed to gather information on Enset landraces, in particular, to assess farmers’ perception of landrace diversity, vernacular naming, folk biosystematics, and description of use values. Interviews were conducted during drinks and coffee times in homes or home gardens, where the selected households and other interested people were gathered together. Because women of rural Gurage zone are particularly responsible for the propagation, protection, harvesting, processing and storage to the final preparation of Enset foods (Tsegaye and Struik, 2002) they were encouraged to participate in the study, and their knowledge, thoughts, and opinions were incorporated. Guided field walks were carried out with the informants in each kebele to obtain essential ethnobotanical information as well as to gather Enset varieties and landraces.

All Enset varieties were collected, dried, and identified, and the voucher specimens were deposited in the National Herbarium of Addis Ababa University. Identification of varieties was made in comparison with authenticated specimens from the herbarium, which were later confirmed by senior taxonomists.

### Data verification and analysis

The collected data were meticulously cross-checked for completeness and reliability. Expert elicitations, key informant comments, and informal discussions with farmer groups were conducted to verify inconsistencies and enrich and validate the information gathered from individual interviews. Descriptive statistical summaries, including frequencies, percentages, ANOVA, and averages, were calculated using SPSS software. Furthermore, the use values of traditional medicinal Enset landraces were calculated following Phillips and Gentry (1993).

## Results and discussion

### Socio-demographic characteristics

The population of the study participants and socio-demographic characteristics is described in Table 2. The table shows the distribution of 603 respondents across four Woredas. There was no significant difference in the number of respondents (p = 0.947) among the different Woredas, revealing an appropriate representation of respondents.

**TABLE 2:** Socio-demographic characteristics of the respondents.

Variable	Category	Woreda’s of the respondents								Mean%
		Cheha		Enor		Gumer		Ezhe		
		Frequency	Percentage	Frequency	Percentage	Frequency	Percentage	Frequency	Percentage	
Gender	Male	63	47.7%	65	43.9%	67	40.6%	88	55.7%	46.975
	Female	69	52.3%	83	56.1%	98	59.4%	70	44.3%	53.025
Age of the respondents	18–30	81	61.4%	110	74.3%	83	50.3%	83	52.5%	59.625
	31–45	37	28.0%	28	18.9%	51	30.9%	67	42.4%	30.05
	46–60	13	9.8%	9	6.1%	12	7.3%	7	4.4%	6.9
	Above 60	1	0.8%	1	0.7%	19	11.5%	1	0.6%	3.4
Marital status	Single	21	15.9%	24	16.2%	7	4.2%	5	3.2%	9.875
	Married	103	78.0%	108	73.0%	153	92.7%	147	93.0%	84.175
	Divorced	3	2.3%	5	3.4%	1	0.6%	4	2.5%	2.2
	widowed	5	3.8%	11	7.4%	4	2.4%	2	1.3%	3.725
Religion	Orthodox	89	67.4%	56	37.8%	48	29.1%	106	67.1%	50.35
	Muslim	25	18.9%	77	52.0%	83	50.3%	39	24.7%	36.475
	Catholic	4	3.0%	11	7.4%	0	0.0%	1	0.6%	2.75
	Protestant	14	10.6%	4	2.7%	34	20.6%	12	7.6%	10.375
Education status	Not read and write	44	33.3%	76	51.4%	77	46.7%	20	12.7%	36.025
	Read only	1	0.8%	1	0.7%	5	3.0%	1	0.6%	1.275
	Read and write	5	3.8%	13	8.8%	36	21.8%	29	18.4%	13.2
	Grade 1–6	19	14.4%	10	6.8%	41	24.8%	22	13.9%	14.975
	Grade 7–8	21	15.9%	19	12.8%	4	2.4%	32	20.3%	12.85
	Grade 9–12	25	18.9%	21	14.2%	2	1.2%	26	16.5%	12.7
	College or university	17	12.9%	8	5.4%	0	0.0%	28	17.7%	9
Occupation (work status)	Government employee	18	13.6%	7	4.7%	2	1.2%	33	20.9%	10.1
	merchant	24	18.2%	23	15.5%	7	4.2%	15	9.5%	11.85
	farmer	66	50.0%	112	75.7%	154	93.3%	101	63.9%	70.725
	Artesian	3	2.3%	0	0.0%	0	0.0%	4	2.5%	1.2
	unemployed with no regular income	15	11.4%	4	2.7%	0	0.0%	4	2.5%	4.15
	retired	0	0.0%	0	0.0%	1	0.6%	1	0.6%	0.3
	Traditional healer	0	0.0%	1	0.7%	0	0.0%	0	0.0%	0.175
	others	6	4.5%	1	0.7%	1	0.6%	0	0.0%	1.45

The demographic data of respondents also revealed distinct variations in age groups, religious affiliations, and occupational and educational attainment (Table 2). However, the distribution concerning gender showed insignificant variation (p = 0.87), which contrasts some ethnobotanical studies conducted in Ethiopia (Assefa and Fitamo, 2016; Tamene et al., 2024; Awoke et al., 2024). The good representation of female in the current study could probably arise from the strong connection that females had with Enset in their daily lives, and thus believed to be knowledgeable about Enset cultivation, harvesting, and using Enset products. They rely on Enset products for most of their food needs, medical requirements, need for fodder, and environmental makeup. The age distribution shows that Enor has the largest proportion of young adults aged 18–30 years, while Gumer has a significant number of elderly respondents above 60 years (Table 2). In terms of religious affiliations, Orthodox (50.35%) is predominant, being followed by Muslims (36.475%). Educational attainment varies significantly among the Woredas, reflecting different levels of access to education. Gumer and Enor have a high percentage of respondents who are illiterate or have only basic reading skills, whereas Cheha and Ezhe have higher proportions of respondents with secondary and tertiary education. Ezhe stands out with the highest percentage of college or university graduates (Table 2).

The occupation or work status of the respondents revealed that the majority (71.8%) of them were farmers, followed by 11.4% and 10% merchants and government employees, respectively. Conversely, about 0.2% of the study participants were traditional healers of bone settlers.

On the other hand, the number of landraces recorded in this study was less than the landraces recorded in Sidama (Tesfaye, 2008), Wolaita (Olango et al., 2014), and Hadiya (Dilebo et al., 2024). This could be attributed to the variations in agro ecology and adaptations of the varieties.

### Indigenous knowledge systems of Enset diversity

A significant finding from the survey is the extent of respondents’ knowledge about Enset varieties. A majority, 57.7%, reported knowing 6–10 varieties, while 21% stated they are familiar with 11–15 varieties, and 5.2% reported more than 15 varieties. The survey conducted thus provides a comprehensive understanding of the knowledge of Enset varieties among respondents. This breadth of knowledge underscores the importance of Enset in local agricultural practices and cultural traditions. The ability to distinguish between varieties ensures that the right type of Enset is used for the right purpose, thereby maximizing its utility. Moreover, as depicted by the key informants, the Gurage zone farmers are extremely knowledgeable about the diversity and agricultural practices of their Enset crop. The unique indigenous knowledge that the community has created over time through interaction with Enset and its farming system and empirical observation is demonstrated by 1) the use of folk biosystematics for maintaining intra-specific Enset diversity, 2) the intricate and overlapping uses derived from Enset landraces, and 3) the dynamic on-farm management practices that preserve landrace diversity. According to every respondent, this indigenous knowledge system was passed down from their ancestors and has been a custom since the beginning of time (Olango et al., 2014).

### Indigenous biosystematics of Enset

**FIGURE 3:**
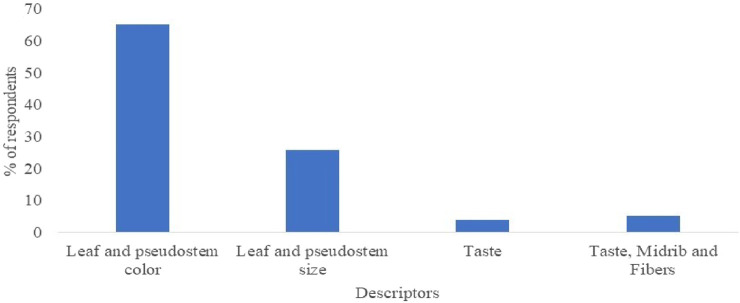
Descriptors used by the Gurage community to identify Enset landraces, south-central Ethiopia.

The local farmers of the four selected study areas of the Gurage zone (Gumer, Cheha, Enor, and Ezeh) remark each Enset landrace they grow as distinct, with clearly distinguishable peculiar characteristics. The farmers used folk processes of indigenous biosystematics for their landraces under cultivation for identification. These descriptors were commonly related to morphological characteristics including pseudostem color, midrib color, and petiole patches/strips colors. Based on the landraces cultivated in the area, the most frequently mentioned (62%) descriptors for identification were leaf color, followed by the size of the leaf and pseudostem color (24.7%) (Figure 3). Leaf color was thus used as a principal descriptor for the identification of Enset landraces by the local people in the study area. Similar morphological characteristics were also used by the local communities of Angacha district, Welene district (Abdella, 2016), and Hadiya zone (Dilebo et al., 2024) of Ethiopia as the primary criterion for the identification and classification of Enset landraces. This traditional knowledge is essential for selecting and cultivating the right Enset varieties for specific purposes, enhancing the plant’s effectiveness and benefits.

### Nomenclature and classification of enset landraces

The study found a total of 37 Enset landraces listed by the respondents (Table 3). Local farmers in the study area give separate vernacular names for each landrace they cultivate. The names are often descriptive and reflect variations of landraces in places of origin, morphology, as well as agronomy and uses. The respondents used the local language, which is predominantly Gurage language, to describe the specific morphological, agronomic, and uses attributes of specific landraces. For instance, the Ensete landrace Astara literally refers to “specific,” Kibinar refers to “soft or gentle” and Dere refers to “common (widespread).” Moreover, the selected four woredas of the Gurage zone (Gumer, Cheha, Ezhe, and Enor) use traditional classification systems for their Enset landraces. The documented Enset landrace diversity in the current study is greater than that of Adola Rede district (Mengesha et al., 2022) but less than that of Sidama Zone (Assefa and Fitamo, 2016), Enemorina Eaner district (Fikadu et al., 2022), and Kembata (Maryo et al., 2018). The variations in the diversity of Enset landraces among different regions might be attributed to characters related to growth and adaptation, climatic variations, accessibility of germplasm, or cultural history, and the level of reliance on Enset as a food.

**TABLE 3:** Vernacular names of Enset landrace in the four woredas of Gurage zone (Gumer, Enor, Cheha and Ezeh) along with distribution from the study report and agroecology.

S. No	Vernacular name	Voucher No	Distribution (study reports)	Distribution (agroecology)
1	Agade	AH09	Medium	H, M, L
2	Amerat	AH12	Medium	H, M, L
3	Ameratiye	AH04	Medium	H, M, L
4	Ankefuye	AH11	Medium	H, M, L
5	Astara	AH01	Common	H, M, L
6	Badedet	AH10	Not Indicated	H, M, L
7	Bazeriye	AH23	Not Indicated	H, M, L
8	Bosere	AH33	Not Indicated	H, M, L
9	Bukiret	AH30	Not Indicated	H, M, L
10	Bore	AH18	Not Indicated	H, M, L
11	Chehuyet	AH16	Not Indicated	H, M, L
12	Dere	AH20	Common	H, M, L
13	Dirbo	AH35	Not Indicated	H, M, L
14	Ehireye	AH17	Not Indicated	H, M, L
15	Emiriye	AH22	Not Indicated	H, M, L
16	Enba	AH21	Not Indicated	H, M, L
17	Fereziye	AH28	Not Indicated	H, M, L
18	Gazinar	AH36	Not Indicated	H, M, L
19	Guareye	AH19	Common	H, M, L
20	Kanchiwo	AH27	Not Indicated	H, M, L
21	Kibnar	AH02	Common	H, M, L
22	Mariye	AH06	Not Indicated	H, M, L
23	Nechewo	AH14	Not Indicated	H, M, L
24	Reziye	AH31	Not Indicated	H, M, L
25	Sapara	AH07	Not Indicated	H, M, L
26	Shiretiye	AH03	Not Indicated	H, M, L
27	Sibisa Sibir	AH13	Not Indicated	H, M, L
28	Sinewot	AH37	Not Indicated	H, M, L
29	Tegadede	AH05	Not Indicated	H, M, L
30	Teriye	AH26	Not Indicated	H, M, L
31	Wosere	AH24	Not Indicated	H, M, L
32	Yefereziye	AH25	Not Indicated	H, M, L
33	Yegodeset	AH34	Not Indicated	H, M, L
34	Yekecheriye	AH32	Not Indicated	H, M, L
35	Yeshirekinke	AH08	Not Indicated	H, M, L
36	Yiregiye	AH15	Not Indicated	H, M, L
37	Zerebadet	AH29	Not Indicated	H, M, L

^*^H= high, M = medium, L = low.

### Indigenous knowledge of the medicinal uses of enset

**FIGURE 4:**
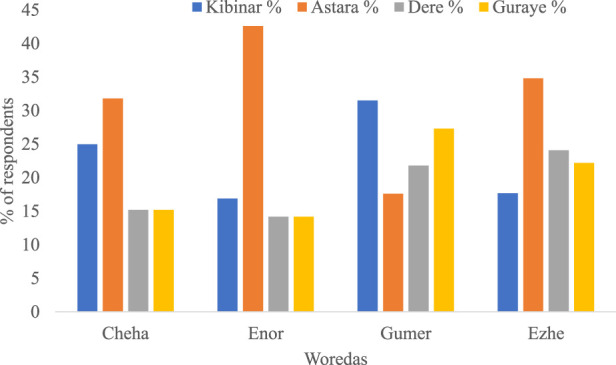
Varieties of Enset used for medicine in four Woredas of Gurage Zone.

Computation of the use value index of the four medicinal Enset landraces revealed that Astara had the highest use value (0.98) demonstrating its potential importance for healing bones among the Gurage community followed by Kibinar (0.72), Guarye (0.62), and Dere (0.6). However, there are variations in the use of Enset varieties as a medicinal plant across different woredas of the study region (Figure 4). In Cheha woreda, the Astara variety is the most commonly used for medicinal purposes (31.8%), while in Enor Woreda, Astara is even more predominant (42.6%). Gumer Woreda shows a higher preference for Kibinar (31.5%), and in Ezhe Woreda, Astara again leads with 34.8% (Figure 4). Overall, except for Gumer Woreda, Astara was the most preferred Enset variety for treating bone fractures. The variations in the use of Enset varieties for medicinal purposes among the Woredas highlight the adaptability of Enset to different local conditions and cultural practices, demonstrating its widespread acceptance and significance. The common utilization of Astara and Kibinar for bone fracture treatment is consistent with the previous study conducted in the zone (Sahle et al., 2021) Moreover, Astara and Kibinar varieties were also reported as the best varieties in the Welene district (Abdella, 2016). These varieties play a crucial role in traditional healing practices for treating broken bones and bone fractures. This role of Enset landraces in healing fractured bones and setting broken bones arises from its mineral contents like calcium (Debebe et al., 2012). The reliance on Enset for such critical health issues highlights the plant’s significance in traditional medicine and its potential as an accessible healthcare resource in these communities.

**FIGURE 5:**
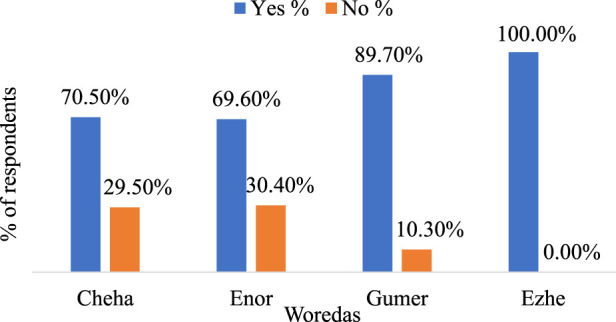
The proportion of Enset varieties used for bone healing or setting of broken or fractured bone in four Woredas of Gurage Zone.

Respondents from different Woredas also indicated varying degrees of reliance on Enset for healing broken bones and fractures (Figure 5). In Cheha, 70.5% of respondents use Enset for this purpose, whereas in Enor, the Figure is 69.6%. Gumer sees a higher rate at 89.8%, and in Ezhe, it is universally acknowledged at 100% (Figure 5). This demonstrates the crucial role of Enset in traditional medicine across different Woredas, showcasing its effectiveness and trust within these communities.

### Parts used and mode of preparation of enset for medicinal purpose

The study revealed that most of the respondents (87.2%) use corm as a major part of the Enset for healing broken or fractured bone (Table 4) while 9.1% use pseudostem for the treatment. Moreover, elucidation from the focus group discussion revealed that the age of 2–3 years (age after being transplanted to a permanent field after being raised for 2 years in a nursery) of Enset landraces was considered optimal for utilizing it in traditional healing practices related to bone injuries, fractures, and setting broken bones. Preference of Enset landraces at this age for bone treatment might be associated with the accumulation of sufficient concentration of beneficial nutrients such as Calcium, Phosphorus, Zinc, and proteins (Atlabachew and Chandravanshi, 2008; Nuraga et al., 2019; Borrell et al., 2020). Nuraga et al. (2019) found 97.3–114.3 mg g^−2^ Calcium, Phosphorus 94.8–127.4 mg g^−2^, Zinc 4.5–8.5 mg g^−2^, and protein 2.4–4.7 mg g^−2^ for similar aged Enset landraces. Calcium plays a potential role in the mineralization of fracture-callus and bone repair (Fischer et al., 2018). Besides Calcium, the presence of Phosphorus as part of phosphate in Enset is vital for healing fractured bones (Wigner et al., 2010). Moreover, studies also reported the potential role of Zinc (O'Connor et al., 2020) and proteins (Pountos et al., 2016) in bone healing. The high amount of arginine obtained from the analysis of the free amino acids of Enset landraces reveals the potential medicinal characteristics of the plant since arginine is related to the formation of collagen, repairing of tissues like broken bones, and healing of wounds through proline (Tamrat et al., 2020). L-arginine reduces bone loss and enhances osteogenesis (Shen et al., 2024). However, further mineral compositional studies concerning different aged Enset landraces are required for verification of the exact concentration level of these minerals since *E. ventricosum* landraces have an average lifespan of 5–10 years (and more in some) as revealed by respondents.

**TABLE 4:** Enset parts used for setting bones.

Enset parts used for healing broken or fractured bone	% Of respondents
Corm	87.2
Leaves	3.3
Pseudostem	9.1
Leaves and pseudostem	0.3

For the treatment of bone fractures and setting broken bones, the Enset landraces as food are indeed consumed orally. The mode of preparation for medicinal purposes typically employs similar methods among the four Woredas involving mixing the boiled Astara and Kibinar varieties (*amicho*) with yogurt, milk, or meat to treat bone fractures and broken bones as elucidated by the different focus group discussions. The key informants revealed that the preparation of these varieties for the treatment of bones is simple since it involves taking the inner part of the corm and boiling it and then eating it like a potato. For effective treatment, equal amounts of Astara and Kibinar landraces must be taken with yogurt or milk rather than taking individual varieties. Similar ingredients were used in Sidama (Assefa and Fitamo, 2016), and Kembata (Ayenew et al., 2016) ethnic groups of Ethiopia. After initial treatment with these varieties, Guarye and Dere is also consumed to strengthen bones, being mixed with similar ingredients. However, it is important to note that Guarye and Dere are not administered directly to pregnant women beyond 5 months of pregnancy, indicating an awareness of specific health considerations in traditional practices. This practice ensures the safety of pregnant women while still utilizing the medicinal benefits of Enset.

### Other uses of Enset

**FIGURE 6:**
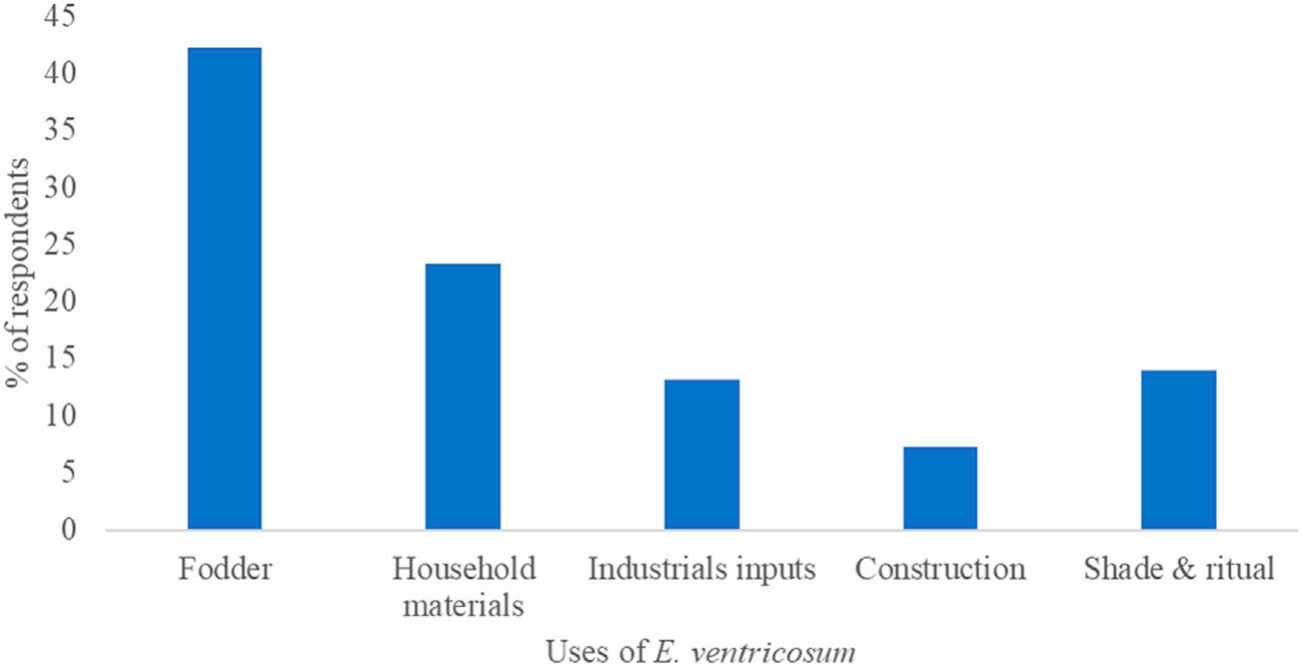
Utilization of Enset other than medicine in Gurage zone.

Beyond its medicinal applications, Enset is also highly valued for its utility in agriculture and household needs. According to the survey, 42.3% of respondents use Enset as fodder for livestock, which supports animal husbandry, a vital component of rural livelihoods (Figure 6). Additionally, 23.3% of respondents use Enset to make household materials twist ropes, and pads, demonstrating its versatility. These findings are consistent with previous research (Tilahun, 2017).

The fact that 91.4% of respondents cultivate Enset in their home gardens (Table 5) further emphasizes its importance as a staple crop that provides food security and multiple utilities for rural households. Similar findings were reported from southern Ethiopia (Maryo et al., 2014; Garedew et al., 2017).

**TABLE 5:** Sites of Enset cultivation in the four Woredas of Gurage Zone, Southcentral region, Central Ethiopia.

Site of enset cultivation	% Of respondents
Home garden	91.4
Backyard	7.3
Natural habitat	0.7
Backyard and natural habitat	0.7

In conclusion, the survey underscores the critical role of Enset in the surveyed communities, highlighting its multifaceted uses from medicinal applications to agricultural and household utilities. The extensive knowledge about different Enset varieties and their specific uses reflects a deep cultural integration and reliance on this plant. Understanding these practices is essential for supporting sustainable agricultural systems and preserving traditional knowledge that is vital for the wellbeing of these communities. The data provides a valuable foundation for further research and development efforts aimed at enhancing the benefits of Enset in these and similar communities.

### Indigenous management and threats to Enset diversity

The indigenous cultivation of Enset landraces in home gardens and backyards by intercropping with other crops was the major means of maintaining the landraces as revealed from FGD in the four woredas. However, key informants reported that the abundance of the different landraces varies among localities based on the preferences associated with yield and quality. Farmers generally favor cultivars with high kocho and bulla yield and quality, which aligns with other findings (Dejene and Yemataw, 2018).

**FIGURE 7:**
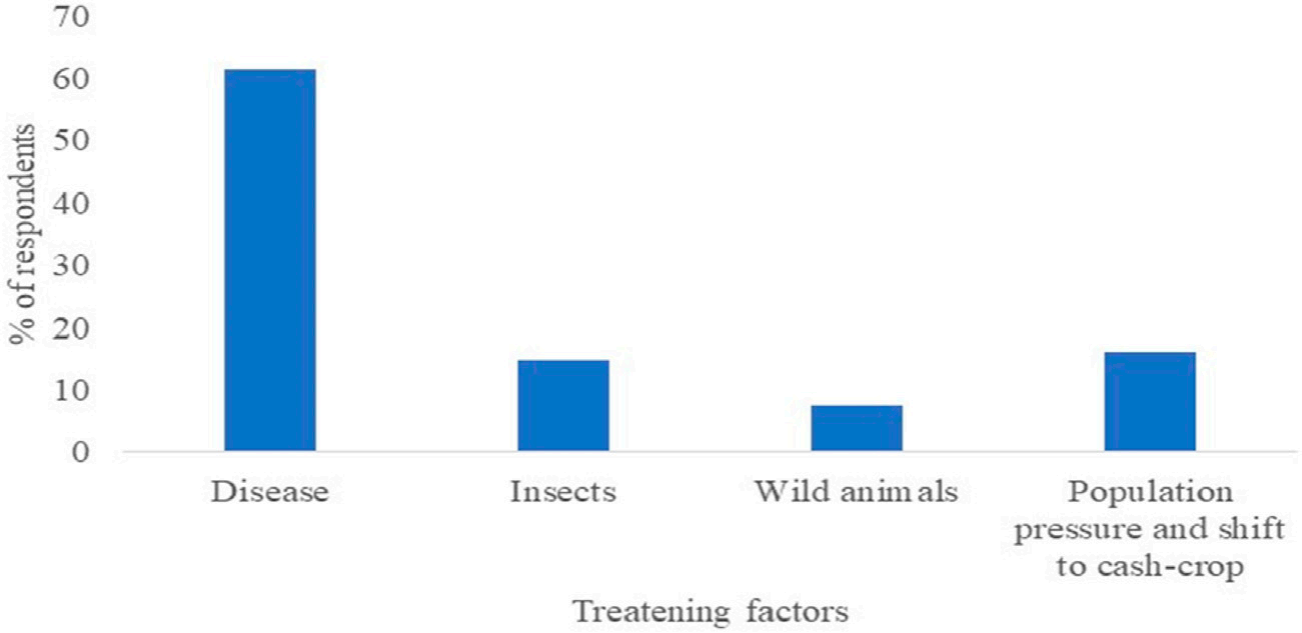
Factors threatening Enset cultivation in Gurage zone.

Disease was reported as the main threatening factor for Enset cultivation in the Gurage zone as revealed by many respondents (30.3%). The most prevalent disease in the area was bacterial wilt which significantly affects Enset production. Moreover, insects (common ants), wild animals (Monkeys, apes, porcupines, wild pigs, and wild boar), and diseases together perceived (51.9%) as the major threatening factors for Enset production in the Gurage zone (Figure 7). Diseases like bacterial wilt and animals were also reported as the main threatening factors for Enset production in Enor and Cheha woredas of the Gurage zone by previous studies (Mitiku et al., 2024).

## Conclusion

Ensete (*Ensete ventricosum*) is a crucial crop for food, medicine, and other purposes in Gumer, Cheha, Enor, and Ezhe woredas of the Gurage zone in Central Ethiopia. The survey conducted provides a comprehensive understanding of the knowledge and utilization of Enset varieties among respondents, revealing the deep integration of this plant into various aspects of daily life, particularly in medicinal and household contexts. Accordingly, a total of 37 Enset landraces were recorded in the four woredas. Local farmers give separate vernacular names (using the Gurage language) which are more descriptive types for the landraces they cultivate that reflect variations of landraces in places of origin, morphology, as well as agronomy and uses. Most farmers reported 6–10 landraces, ensuring their potential ability to identify and differentiate between various Enset types, underscoring the importance of Enset in local agricultural practices and cultural traditions. The farmers used folk processes of indigenous biosystematics for their landrace under cultivation for identification. Leaf color was the most frequently mentioned descriptor for identification of the landraces followed by the size of the leaf and pseudostem color.

Respondents from the study Woredas indicated that they rely on Enset for healing fractured and broken bones. Astara variety is the most commonly used variety to treat bone fractures and setting broken bones in all study Woredas of the Gurage zone. In addition, Kibinar, Dere, and Guraye were also commonly used for bone treatment in the Woredas. The major part of Enset used for healing fractured and broken bones was the corms at the age of 2–3 years. The mode of preparation for bone treatment involves mixing Astara and Kibinar varieties with yogurt, milk, or meat to treat bone fractures as elucidated by the different focus group discussions. Besides food and medicinal applications, Enset has been used as fodder and household materials in the study area.

Most of the cultivation of Enset landraces in the four Woredas was carried out in home gardens followed by backyards. However, diseases were found to be the major threatening factors for Enset cultivation followed by insects and animals in the study area requiring effective conservation strategies that integrate indigenous knowledge and scientific methods. To ensure the sustainable use and preservation of Enset varieties, it is crucial to increase educational initiatives within local communities, disseminating comprehensive knowledge about their diverse applications. Although the study found that the community in the study area has been using the four Enset landraces (Astara, Kibinar, Guarye, and Dere) for many years to heal fractured and broken bones, validating them has been challenging. Thus, traditional healing practices utilizing Enset should be supported by integrating them with modern healthcare systems, providing resources, and validating their benefits in official health policies. Advocating for the cultivation of multiple Enset varieties will enhance food security and agricultural resilience, supported by subsidies and quality planting materials, supported by the government. Additionally, investing in scientific research to validate Enset’s medicinal properties will facilitate the development of new treatments, benefiting both local and global communities. Verifying the exact concentration of minerals in the medicinal Enset landraces used in treating bones and phylogenetic profiling of these varieties are crucial in future research.

## Data Availability

The raw data supporting the conclusions of this article will be made available by the authors, without undue reservation.
